# Considerations for target oxygen saturation in COVID-19 patients: are we under-shooting?

**DOI:** 10.1186/s12916-020-01735-2

**Published:** 2020-08-19

**Authors:** Niraj Shenoy, Rebecca Luchtel, Perminder Gulani

**Affiliations:** 1 Department of Medicine (Oncology), Albert Einstein College of Medicine, Montefiore Medical Center, 1300 Morris Park Avenue, Bronx, NY 10461 USA; 2 Department of Medicine (Critical Care Medicine), Albert Einstein College of Medicine, Jacobi Medical Center, Bronx, NY 10461 USA

**Keywords:** SARS-CoV-2, COVID-19, Hypoxemia, Hypoxia, ACE2

## Abstract

**Background:**

The current target oxygen saturation range for patients with COVID-19 recommended by the National Institutes of Health is 92–96%**.**

**Main body:**

This article critically examines the evidence guiding current target oxygen saturation recommendation for COVID-19 patients, and raises important concerns in the extrapolation of data from the two studies stated to be guiding the recommendation. Next, it examines the influence of hypoxia on upregulation of ACE2 (target receptor for SARS-CoV-2 entry) expression, with supporting transcriptomic analysis of a publicly available gene expression profile dataset of human renal proximal tubular epithelial cells cultured in normoxic or hypoxic conditions. Finally, it discusses potential implications of specific clinical observations and considerations in COVID-19 patients on target oxygen saturation, such as diffuse systemic endothelitis and microthrombi playing an important pathogenic role in the wide range of systemic manifestations, exacerbation of hypoxic pulmonary vasoconstriction in the setting of pulmonary vascular endothelitis/microthrombi, the phenomenon of “silent hypoxemia” with some patients presenting to the hospital with severe hypoxemia disproportional to symptoms, and overburdened health systems and public health resources in many parts of the world with adverse implications on outpatient monitoring and early institution of oxygen supplementation.

**Conclusions:**

The above factors and analyses, put together, call for an urgent exploration and re-evaluation of target oxygen saturation in COVID-19 patients, both in the inpatient and outpatient settings. Until data from such trials become available, where possible, it may be prudent to target an oxygen saturation at least at the upper end of the recommended 92–96% range in COVID-19 patients both in the inpatient and outpatient settings (in patients that are normoxemic at pre-COVID baseline). Home pulse oximetry, tele-monitoring, and earlier institution of oxygen supplementation for hypoxemic COVID-19 outpatients could be beneficial, where public health resources allow for their implementation.

## Background

The current target oxygen saturation range for patients with COVID-19 recommended by the NIH is 92–96%. “The use of supplemental oxygen in adults with COVID-19 has not been studied, but indirect evidence from other critical illnesses suggests the optimal oxygen target is an SpO_2_ between 92% and 96%” (https://www.covid19treatmentguidelines.nih.gov/critical-care/oxygenation-and-ventilation/). The indirect evidence refers to the following two studies:

- A meta-analysis of 25 RCTs (randomized controlled trials) in 16,037 acutely ill patients [1], which concluded that liberal oxygenation (median 96%, range 94–99%) was associated with increased mortality (relative risk 1·21, 95% CI 1·03–1·43) when compared with conservative oxygenation.
- The LOCO-2 trial [2] where ARDS (acute respiratory distress syndrome) patients were randomized to conservative (target partial pressure of arterial oxygen [PaO_2_], 55 to 70 mmHg; oxygen saturation as measured by pulse oximetry [SpO_2_], 88–92%) vs liberal (target PaO_2_, 90 to 105 mmHg; SpO_2_, ≥ 96%) oxygen arms. The trial was stopped early due to increased deaths in the conservative arm. At day 90, 44.4% of patients in the conservative-oxygen group and 30.4% of patients in the liberal-oxygen group had died (difference, 14.0 percentage points; 95% CI, 0.7 to 27.2).

## Main body

Here, we examine the above two studies guiding current target oxygen saturation recommendations for COVID-19; discuss, with supporting transcriptomic analyses, the influence of hypoxia on ACE2 (angiotensin converting enzyme-2, target receptor for SARS-CoV-2 entry) expression; reflect on relevant clinical observations and considerations in COVID-19 patients; and propose a re-evaluation of target oxygen saturation in these patients—both in the inpatient and outpatient settings.

### Critical analysis of studies guiding current target oxygen saturation recommendation

*First*, a closer look at the two studies on which the current recommendations are based:

The 2018 meta-analysis was not specific to ARDS (or even hypoxemia). RCTs in non-hypoxemic stroke patients exploring supplemental oxygen vs room air were included in the analysis, with supplemental oxygen being grouped in the overall “liberal oxygenation” arm and room-air oxygenation in non-hypoxemic patients grouped under the overall “conservative oxygenation” arm. Non-hypoxemic stroke patients receiving room air, i.e., “conservative oxygenation,” had a lower death rate. Similarly, RCTs of supplemental oxygen vs room air in largely normoxemic patients with myocardial infarction were also included in the analyses. Extrapolating these data to patients with ARDS raises significant concerns of relevance. Next, one of the RCTs included in the meta-analysis, the Oxygen-ICU Randomized Clinical Trial in critically ill patients [3], had a significant influence on the final analysis with a death rate of 80/243 vs 58/235 in liberal vs conservative oxygenation. In that study, however, “conservative oxygenation” was defined as an SpO_2_ of 94–98% or PaO_2_ between 70 and 100 mmHg, whereas conventional/liberal oxygenation was defined as an SpO_2_ of 97–100%, allowing PaO_2_ values up to 150 mmHg [3]. Therefore, what was considered “conservative” in that study had overlapping saturation ranges with the definition of “liberal” in the overall analysis. In addition, patients in the “liberal” arm in that study were allowed very high non-physiologic PaO_2_ levels.

Prior to the LOCO-2 trial, the National Heart, Lung, and Blood Institute ARDS Clinical Trials Network recommended a target PaO_2_ between 55 and 80 mmHg (SpO_2_ 88–95%). In fact, the LOCO-2 trial was conducted with the hypothesis that the lower limits of that range (PaO_2_ between 55 and 70 mmHg) would improve outcomes in comparison with target PaO_2_ between 90 and 105 mmHg. The opposite was true (adjusted hazard ratio for 90-day mortality of 1.62; 95% CI 1.02 to 2.56), and the trial was stopped early. Five mesenteric ischemic events were reported in the conservative-oxygen group.

Put together, RCT data in ARDS patients evaluating target SpO_2_ ≥ 96% (with a target upper PaO_2_ limit of 105 mmHg) vs target SpO_2_ 92–95% are lacking. RCT data in ARDS has demonstrated that SpO_2_ ≥ 96% is significantly better than SpO_2_ 88–92%. Basing oxygen saturation recommendations in ARDS patients, in part, on the 2018 meta-analysis, raises important concerns as detailed above.

### ACE2 and hypoxia

*Second*, the role of ACE2 in SARS-CoV-2 pathogenesis and progression as a target receptor for viral entry as well as the influence of hypoxia on ACE2 expression merits particular consideration. ACE2 is a negative regulator of the angiotensin system and a counter-regulatory enzyme of ACE. While ACE coverts angiotensin I to angiotensin II, ACE2 degrades angiotensin II to angiotensin-(1-7). ACE2 expression and its catalytic product angiotensin-(1-7) have been shown to be protective against lung injury and ARDS by opposing the proliferative, hypertrophic, and fibrotic effects of angiotensin II [4–10].

SARS-CoV-2, by targeting (using as an entry receptor) the very protein that is protective against the above deleterious effects, poses unique challenges. The binding affinity of SARS-CoV-2 Spike protein to ACE2 receptor has been reported to be 10–20 times higher than that with SARS-CoV Spike protein [11], likely playing a key role in the markedly enhanced virulence. *ACE2* knockout mice had significantly lower lung injury scores and SARS-CoV Spike RNA from SARS-CoV infection compared to wild type [12].

In humans, ACE2 is expressed abundantly on the surface of lung alveolar epithelial cells and enterocytes. It is also expressed in arterial and venous endothelial cells as well as arterial smooth muscle cells within multiple organs (lung, stomach, intestines, kidney, brain, bone marrow, spleen, etc.) [13]. This widespread expression of ACE2, and its high affinity with the SARS-CoV-2 Spike protein, possibly accounts for the range of severe clinical manifestations apart from ARDS, including acute renal failure and encephalopathy, with the pathogenic mechanism being diffuse endothelitis and microthrombi [14–16].

**Fig. 1:**
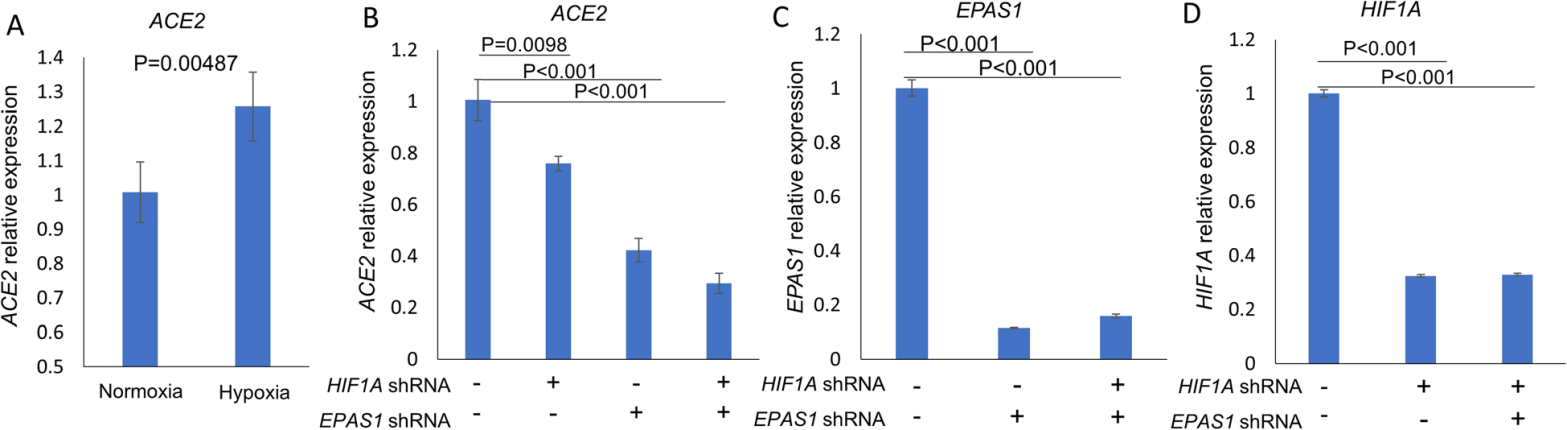
**a** Human renal proximal tubular epithelial (HK2) cells were cultured simultaneously under either normoxic (20% O_2_) or hypoxic (1% O_2_) conditions for 24 h. Hypoxia increased *ACE2* expression (**b**–**d**). HK2 cells stably expressing shRNA (short hairpin RNA) targeting *HIF1A* and/or *EPAS1* were cultured under hypoxic (1% O_2_) conditions for 24 h. (**b**). Under hypoxic conditions, knockdown of *EPAS1* and *HIF1A*, alone and in combination, reduced *ACE2* expression (**c**, **d**). shRNA knockdown of *EPAS1* and *HIF1A* gene expression was confirmed. Data expressed as mean ± SE, with 3 replicates per group (*n* = 3) [19]. The gene expression profile of harvested cells was analyzed by microarray. Data were accessed through the Gene Expression Omnibus, GSE99324, and processed using affy and limma packages [20–22]. [*In summary*, hypoxia increased expression of *ACE2* transcript in human renal proximal tubular epithelial (HK2) cells. Knockdown of hypoxia inducible factors 1A and 2A (encoded by *HIF1A* and *EPAS1*) with shRNA in hypoxic HK2 cells reduced *ACE2* transcript, indicating that hypoxia-induced upregulation of *ACE2* transcript in these cells is likely mediated through the hypoxia inducible factors. Hypoxia➔ ↑HIF1A and ↑HIF2A ➔ ↑*ACE2*] [Abbreviations: HIF1A, hypoxia inducible factor-1-alpha; EPAS1, endothelial PAS domain-containing protein 1; GEO, Gene Expression Omnibus; shRNA, short hairpin RNA—artificial RNA molecule with a tight hairpin turn that can be used to silence target gene expression via RNA interference (RNAi)]

Intriguingly, pulmonary artery smooth muscle cells (PASMC) in rats have been shown to increase the expression of ACE2 with hypoxia, both at the transcript and protein levels [17]. In the experiment, the cells were incubated at 3% oxygen concentration for 0, 6, 12, 24, and 48 h. The normalized *ACE2* transcript reached a maximum of 3-fold at the 12-h timepoint, and the normalized ACE2 protein expression reached a maximum of 2-fold at the 24-h timepoint, both with high statistical significance (Fig. 1C, 1D of ref. [17]). Similar effect of hypoxia on upregulation of ACE2 expression, both at the transcript and protein levels, has also been demonstrated in human pulmonary artery smooth muscle cells (Fig. 1A-E of ref. [18]).

We therefore sought to determine if the same trend could also be observed in other human cells, by analyzing transcriptomic datasets deposited in Gene Expression Omnibus (GEO). Indeed, we found that human renal proximal tubular epithelial (HK2) cells cultured in hypoxic conditions for 24 h had an increase in the *ACE2* transcript (raw *p* value = 0.0048, adjusted *p* value < 0.05, Fig. 1a) [19]. Furthermore, knockdown of hypoxia inducible factors 1A and 2A (encoded by *HIF1A* and *EPAS1*) in hypoxic HK2 cells reduced *ACE2* transcript (Fig. 1b–d) [19], indicating that hypoxia-induced upregulation of *ACE2* in these cells is likely mediated through the hypoxia inducible factors.

Put together, cellular hypoxia, via upregulating the target receptor for viral entry, could potentially further contribute to an increase in the severity of SARS-CoV-2 clinical manifestations. This is yet to be tested in an in vivo model or in humans. It may be useful to determine the effect of hypoxemia on soluble ACE2 receptor levels in COVID-19 patients.

### Relevant clinical observations and considerations

*Third*, a few clinical considerations:

Hypoxic pulmonary vasoconstriction is a well-recognized phenomenon [23, 24]. With clinical observations of several COVID-19 patients having a marked hypoxemia disproportional to the degree of infiltrates, pulmonary vasculature endothelitis and microthrombi which were suspected clinically have now been shown to be a prominent feature of COVID-19 lung pathology [25]. Any component of hypoxic pulmonary vasoconstriction and further exacerbation of pulmonary hypertension in this setting is best avoided. Further to this point, nocturnal drop in oxygen saturation is a well-known phenomenon [26], is common in patients with primary pulmonary hypertension [27], and has also been demonstrated in patients with pneumonia and sepsis [28]. Nocturnal hypoxemia could therefore potentially further exacerbate reflex pulmonary vasoconstriction as well as peripheral tissue hypoxia in patients with COVID-19 pneumonia. Patients in regular inpatient wards or at home who maintain an SpO_2_ of 92–94% during the day, with or without O_2_ supplementation, can have nocturnal drops into the 80s, with higher drops in patients with obstructive sleep apnea—a highly prevalent morbidity in obese patients.

Next, diffuse systemic endothelitis and microthrombi play an important pathogenic role in the wide range of systemic manifestations (such as acute renal failure, encephalopathy, cardiovascular complications) seen in COVID-19 patients [14–16, 29], explaining the improved outcomes associated with systemic anticoagulation [29]. In the presence of these systemic microthrombi, hypoxemia would be expected to result in a higher degree of peripheral tissue hypoxia/injury. This is another reason why the optimal oxygen saturation in COVID-19 ARDS may be higher than that in ARDS of other etiologies.

The phenomenon of “silent hypoxemia” resulting in some COVID-19 patients presenting to the hospital with severe hypoxemia disproportional to symptoms is now being increasingly noted [30–32], and albeit not fully understood at this stage, may be a harbinger for clinical deterioration [30], and further supports outpatient monitoring with pulse oximetry and earlier institution of oxygen supplementation.

Lastly, with overburdened health systems around the world and viral transmission considerations, COVID-19 patients in the outpatient setting (suspected and confirmed) are instructed to come in to the hospital if their respiratory status deteriorates, most often with no oxygen saturation monitoring at home. While this approach may be essential in managing burdened health system resources and caring for the critically sick, it risks a significant delay in oxygen supplementation for patients in the outpatient setting. With the lack of strikingly effective therapeutic modalities to date, inpatient mortality numbers and percentages for COVID-19 patients around the world have been staggering [33–37]. (It is of relevance to note here that even in non-COVID-19 pneumonia outpatients, oxygen saturations less than 92% are known to be associated with major adverse events [38].)

Put together, while the effects of the degree/duration of hypoxemia in COVID-19 patients have not been comprehensively studied, the concern of its potential adverse effects (above that in pneumonia/ARDS of other etiologies) is based on the above-detailed specific considerations and well-known principles in respiratory/internal medicine. If maintaining a higher oxygen saturation in hypoxemic COVID-19 patients in the outpatient setting could have a role in decreasing the severity of disease progression and complications, earlier institution of oxygen supplementation at home and tele-monitoring could potentially be beneficial.

## Conclusions

The above considerations, put together, call for an urgent exploration and re-evaluation of target oxygen saturation in COVID-19 patients, both in the inpatient and outpatient settings. While conducting randomized controlled trials in the inpatient setting exploring a target SpO_2_ ≥ 96% (target upper PaO_2_ limit of 105 mmHg) vs target SpO_2_ 92–95% would be relatively less complex in terms of execution and logistics, the outpatient setting would require special considerations such as frequent tele-visits and pulse oximetry recordings, home oxygen supplementation as needed to meet target oxygen saturation, and patient compliance. Until data from such trials become available, it may be prudent to target an oxygen saturation at least at the upper end of the recommended 92–96% range in COVID-19 patients both in the inpatient and outpatient settings (in patients that are normoxemic at pre-COVID baseline). Home pulse oximetry, tele-monitoring, and earlier institution of oxygen supplementation for hypoxemic COVID-19 outpatients could be beneficial but should be studied systematically given the significant public health resource implications.

## Data Availability

Not applicable

## Abbreviations

ACE2: Angiotensin converting enzyme-2
ARDS: Acute respiratory distress syndrome
SpO_2_: Oxygen saturation
PaO_2_: Partial pressure of oxygen
SARS-CoV-2: Severe acute respiratory syndrome coronavirus 2
COVID-19: Coronavirus disease 2019
PASMC: Pulmonary artery smooth muscle cells
HIF1A: Hypoxia inducible factor-1-alpha
EPAS1: Endothelial PAS domain-containing protein 1
RCT: Randomized controlled trial
GEO: Gene Expression Omnibus
shRNA: Short hairpin RNA
